# Spatial disparities of antenatal care utilization among pregnant women in sub-Saharan Africa—Bayesian geo-additive modelling approach

**DOI:** 10.3389/fpubh.2025.1517724

**Published:** 2025-06-27

**Authors:** Denekew Bitew Belay, Haile Mekonnen Fenta, Nigussie Adam Birhan, Najmeh Nakhaei Rad, Ding-Geng Chen

**Affiliations:** ^1^Department of Statistics, Bahir Dar University, Bahir Dar, Ethiopia; ^2^Department of Statistics, University of Pretoria, Pretoria, South Africa; ^3^Center for Environmental and Respiratory Health Research, Population Health, University of Oulu, Oulu, Finland; ^4^Biocenter Oulu, University of Oulu, Oulu, Finland; ^5^Department of Statistics, College of Natural and Computational Science, Injibara University, Injibara, Ethiopia; ^6^College of Health Solution, Arizona State University, Phoenix, AZ, United States

**Keywords:** antenatal care contacts, geo-additive, spatial disparities, INLA, sub-Saharan Africa

## Abstract

**Background:**

Antenatal care (ANC) is critical for ensuring healthy pregnancies and positive birth outcomes. Despite its importance, significant disparities in ANC access and utilization exist across sub-Saharan Africa (SSA), influenced by various socioeconomic, geographical, and systemic factors. This study aimed to analyze the spatial disparities in the proportion of recommended ANC utilization and its associated risk factors among pregnant women in 34 sub-Saharan African countries.

**Method:**

This study utilized the most recent Demographic and Health Survey (DHS) data from 34 countries across the SSA region. To assess the spatial disparities and their associated risk factors of ANC utilization, a geo-additive model via the Integrated Nested Laplace Approximation (INLA) was adopted.

**Result:**

The overall prevalence of recommended ANC utilization in SSA was 22.15%, with a significant difference between countries, ranging from 0.27% in Rwanda to 76.28% in Zimbabwe. Both Moran’s I and Geary’s C tests, with different neighborhood structures, evidenced the existence of spatial autocorrelation of ANC utilization among women in SSA countries. A Bayesian geo-additive model with Besag-York-Mollié (BYM) mixed effect was found to be the best model to assess the spatial dependencies and the non-linear effects of the factors on ANC utilization among women of reproductive age. The study showed that the existence of spatial disparities in ANC utilization and media exposure, as well as the mother’s work status, partner’s working status, age of mother, age at first cohabitation, and place of delivery, has a significant effect on ANC utilization.

**Conclusion:**

The overall coverage of recommended ANC in SSA countries falls short of the global minimum recommended ANC utilization. The lower coverage and inequality of ANC utilizations are influenced by underutilization of healthcare services, economic status, women’s education coverage, poor/absence of transportation facilities, and media exposure related to healthy reproduction. Empowering women through different media outlets, strengthening their economic power, easy access to health facilities, and decision-making power increases maternal healthcare service utilization.

## 1 Background

Improved utilization of maternal healthcare services is an essential prerequisite for early detection of mothers who are at high risk of illness and mortality during pregnancy. Due to low access and utilization of maternal healthcare services, especially in developing countries, women remain vulnerable and underserved. Antenatal care (ANC) is a crucial aspect of healthcare during pregnancy, provided by qualified professionals, and must be initiated within the first 12 weeks of gestation. Recommended follow-up visits help to reduce the risk of stillbirths and pregnancy complications, while also ensuring a positive pregnancy experience for women (1, 2).

Moreover, antenatal care (ANC) serves as an essential gateway for pregnant women to access health promotion and preventive services, including the treatment and management of hypertension and diabetes, iron supplementation, deworming medications, intermittent preventive treatment for malaria, prevention of mother-to-child transmission of HIV, nutritional counselling, and tetanus vaccinations, all of which contribute to healthy pregnancies and positive birth outcomes (3–9).

Pregnant women suffer from direct and indirect complications associated with pregnancy, and related maternal and child mortality is one of the significant global public health challenges facing developing nations (10–13).

Although global maternal mortality decreased by approximately 34% from 2000 to 2020, it is still unacceptably high, and about 287,000 women died during pregnancy and childbirth in 2020. Nearly 800 women die each day from preventable pregnancy and childbirth-related causes, equating to a maternal death every 2 min, of which 94% occur in low- and middle-income countries (11, 14–16).

The recent findings indicate that an increase in the frequency of contacts between a pregnant woman and a skilled healthcare provider can significantly lower maternal mortality rates. The World Health Organization (WHO) has previously recommended that every pregnant woman should attend a minimum of four ANC contacts during a typical pregnancy under normal circumstances (17). However, in 2016, the WHO updated its guidelines to recommend eight or more ANC contacts, with the initial contacts occurring within the first 12 weeks of gestation and subsequent contacts at 20, 26, 30, 34, 36, 38, and 40 weeks. The revision aims to enhance the effectiveness, timing, and quality of care provided by skilled health professionals (4, 11, 18–20).

Globally, 87% of women receive at least one antenatal care (ANC) service from trained health personnel. While the utilization of optimal ANC contacts during pregnancy promotes healthy practices that are proven to reduce maternal mortality and morbidity, and ensure safe motherhood with improved maternal health outcomes, only 60% of women receive the recommended number of ANC contacts throughout their pregnancy (17, 19).

In SSA, 49–53% of women received the recommended ANC visits, and 13% of women had no antenatal care contacts, which can be a significant opportunity for pregnant mothers to mitigate maternal and child mortality and morbidity. This can be achieved directly through identifying and treating pregnancy-related illness, or indirectly through the detection of women at risk of complications of delivery and facilitating their access to adequately equipped healthcare facilities by increasing women’s access to essential obstetric services (18, 21).

Maternal mortality rates are prevalent in certain parts of the world, which underscores regional disparities in access to healthcare. Although timely access to ANC can reduce both direct and indirect causes of maternal mortality, various factors influence the ANC visits, such as maternal age (22), women’s education (22, 23), wealth index (24), ever born children (25), place of residence (26), and distance to health facilities (22, 27).

During a typical pregnancy, 52% of countries worldwide recommend at least four antenatal care (ANC) visits, 39% recommend at least eight visits, and 3% recommend fewer than four. Regionally, there is variation in the adoption of the WHO recommendation for eight ANC visits during a normal pregnancy. In the African region, 48% of countries suggest a minimum of eight visits, while 41% suggest at least four visits (2, 28).

In many parts of sub-Saharan Africa, traditional beliefs and practices further complicate access to recommended ANC services, with some communities favoring traditional birth attendants over formal healthcare providers. Mothers from the same geographical areas often share similar beliefs and culture, which often dictate their common exposure to health-related services, including ANC. Consequently, nations with multiple and diverse cultures are likely to record huge variations in the prevalence of ANC based on their geographic locations (29, 30).

Moreover, the availability and quality of healthcare services also vary widely across sub-Saharan Africa. Inadequate healthcare infrastructure, shortages of trained healthcare personnel, and inconsistent supply of medical resources impede the provision of comprehensive ANC services. Health system inefficiencies, such as poor healthcare governance and a lack of funding, further contribute to the disparities. It is essential to recognize that understanding the geographical variability in recommended ANC and its determinants is vital for improving maternal and child health interventions (31–33).

Despite its importance, significant spatial disparities in ANC access and utilization exist across SSA, influenced by various socioeconomic, geographical, and systemic factors (34, 35). Addressing spatial disparities in ANC utilization in the region requires a multifaceted approach that targets socioeconomic, geographical, and health system barriers. Enhanced policy measures, increased investment in healthcare infrastructure, and targeted community-based interventions are essential to ensure that all women, regardless of their location, have access to vital antenatal care services. As geographic disparities in healthcare services pose a challenge to policymakers and practitioners, assessing the geographical pattern of ANC contacts using the most recent Demographic and Health Survey (DSH) (36) data is critical for planning and implementing geographically focused interventions that will enhance the ANC visits in sub-Saharan African countries. Therefore, this study aims to analyze the spatial distribution and understand disparities in the proportion of recommended antenatal care utilization and the associated factors among pregnant women in 34 sub-Saharan African countries.

This study is structured as follows: it begins with an introduction, which outlines the background, significance, and research gaps related to antenatal care (ANC) utilization across 34 sub-Saharan African countries. The methodology details the study design, data sources, and statistical models, as well as the rationale behind choosing these different models for the analysis. The results section presents key findings on ANC across SSA countries. Moreover, the results section is presented in four subsections: descriptive results, spatial dependency, linear and additive modelling results, and finally, the geo-additive modelling results are presented. The discussion section attempted to contextualize these findings in relation to existing literature, and the conclusion summarizes the results obtained and provides recommendations that may relate to policy implications and possible intervention mechanisms to mitigate the existing disparities.

## 2 Data and method

### 2.1 Data sources and study area

**Figure 1:**
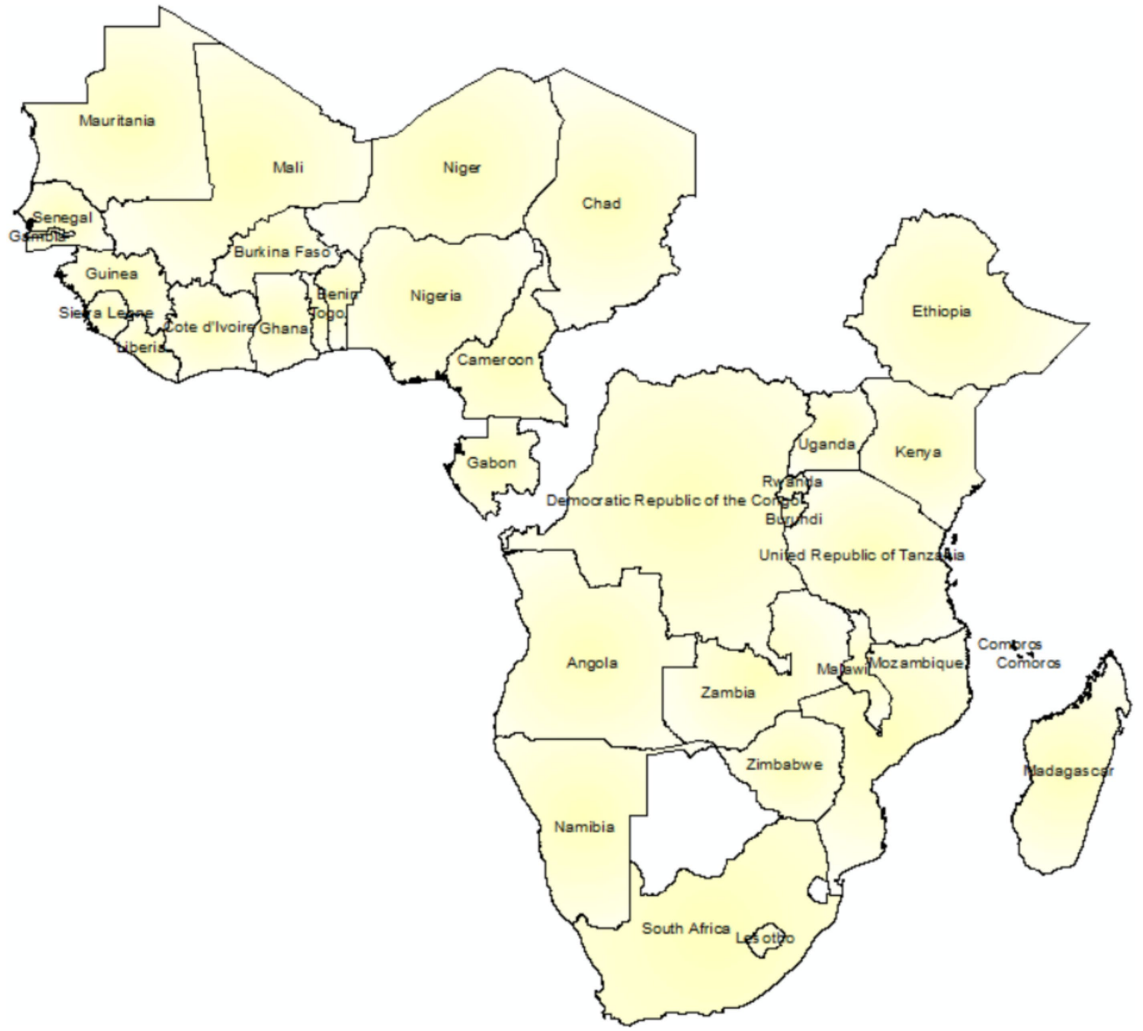
Lists of sub-Saharan African countries considered in this study.

The data used in this study were obtained from a nationally representative recent Demographic and Health Survey data collected by each country in sub-Saharan Africa, conducted between 2010 and 2023. The data is freely available upon reasonable request and can be accessed from https://dhsprogram.com. The shape files for the administrative regions or provinces of each country are also obtained from https://spatialdata.dhsprogram.com/boundaries/#countryId=AO&view=table. The current analysis included women of childbearing age (15–49 years) who had at least one live birth in the last 5 years preceding the time of interview and participated in surveys in 34 sub-Saharan African countries (Figure 1). All records related to ANC visits, which had complete records in all DHS documents, were included in the study. A weighted sample of 223,155 reproductive women from 34 SSA countries is included in this study. A multi-stage stratified sampling technique was used for all Demographic and Health Survey data in low-income countries. The pregnancy and postnatal section of the women’s questionnaire contains information on the use of antenatal care, and women were asked whether they received any antenatal care during pregnancy, with other details, such as the number of ANC contacts they made, and whether they had received prenatal care during their pregnancy (36).

### 2.2 Study variables

#### 2.2.1 Response variable

**Figure 2:**
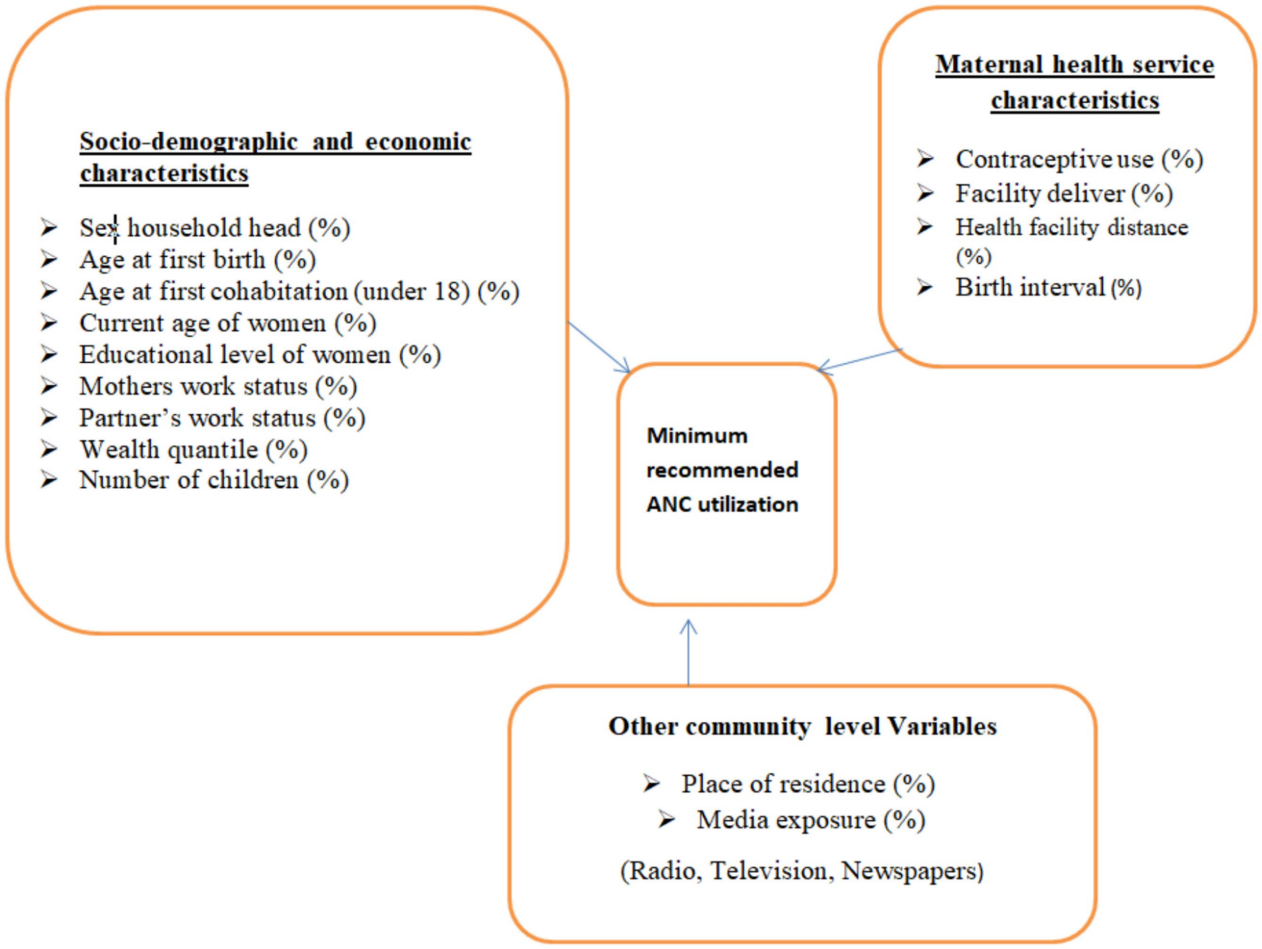
Diagrammatic representation of outcome variables and the respective factors.

The response variable considered in this study was the proportion of a minimum recommended antenatal care contact by trained health professionals. The minimum recommended ANC contacts (a minimum of 4 contacts) were first declared by WHO, and this guideline was used until 2016. Surveys conducted before 2016 (including those before this guideline was established) were considered the gold standard for this study. Then, the WHO revised the minimum recommended ANC contacts to eight again. Surveys conducted thereafter were based on this guideline, requiring a minimum of eight ANC contacts. Therefore, these two recommended WHO guidelines were applied, along with the corresponding surveys to measure the proportion of women meeting the minimum ANC contacts in each region of all SSA countries. Antenatal care contacts with trained health professionals are recommended to have a minimum of contacts, as this can reduce perinatal mortality and improve women’s experience of care (20, 37, 38). The diagrammatic representation of the response variable and the selected potential risk factors is shown in Figure 2.

#### 2.2.2 Risk factors

All factors considered in this study are community-level variables, which are extracted at the regional level by calculating the proportion of each specific category based on the previous literature, and the details of all factors are described in Table 1 (26, 34, 35, 39–41).

**Table 1:** Description of community-level factors/variables.

S. No.	Risk factors	Description
1	Health facility delivery (%)	The proportion of women who delivered at the Health facility in the region
2	Media exposure (%)	The proportion of women who have exposure to the media outlets
3	Partner’s work status (%)	The proportion of women’s partners who have work
4	Mother’s work status (%)	The proportion of women with current work status
5	Wealth quantile (%)	The proportion of women with wealth quantile below the middle wealth quantile (poor and very poor wealth quantile)
6	Place of residence (%)	The proportion of women living in rural residences in the region
7	Contraceptive use (%)	The proportion of women who use any type of contraceptive
8	Number of children (%)	The proportion of women with more than six ever-born children
9	Health facility distance (%)	The proportion of very far health facilities (distance) in the region
10	Educ. level of women (%)	The proportion of educated women in the given region
11	Household head (%)	The proportion of female-headed households in the region
12	Age at first cohabitation (under 18) (%)	The proportion of women who got married below 18 years
13	Current age of women (%)	The proportion of women below the median age
14	Short birth interval	The proportion of women with a short birth interval
15	Survey year	“Yes” (if the survey took place after the WHO’s new guideline 2017 and later), “No” otherwise

### 2.3 Statistical model and data analysis

#### 2.3.1 Data explorations and extraction

Data extraction and descriptive statistics were performed with Stata version 14 (42). In the descriptive analysis, frequencies and percentages are explored using tables and figures. We first apply a linear model and then extend it by relaxing the effect of the risk factor to achieve a parsimonious estimation of each parameter. Then, we extend the linear model to include an additive fixed effect to assess its improvements in predicting the outcome variable again. We also extended the fixed effects additive model to an additive mixed model. Moreover, we tested the existence of spatial autocorrelation by using different spatial dependency tests and neighborhood structure approaches. Finally, by considering the existence of both additive mixed effects of the risk factors on the response variable and the existence of spatial autocorrelation, we applied a geo-additive modelling approach to fit the data to leverage these two data properties in our model fully (43–46).

#### 2.3.2 Spatial autocorrelation

Spatial autocorrelation is a measure of the degree to which a variable or phenomenon is correlated with itself based on its spatial location. It evaluates the orientation and strength of the relationship between a variable and its neighboring values. Global spatial autocorrelation is defined as a measure of the overall clustering of data, providing a single correlation statistic to summarize the entire study area (47). In this study, we employed the Global Moran’s I and Geary’s C statistics to evaluate the spatial dependency of ANC utilization among women across SSA countries. Spatial autocorrelation is also used to detect the spatial autocorrelation of ANC visits. Calculated Moran’s I values close to −1 indicate it is dispersion, whereas I values close to +1 indicate clustering, and a random distribution is indicated if the I value is zero. A statistically significant Moran’s I (*p* < 0.05) leads to rejection of the null hypothesis (spatial independence) and indicates the presence of spatial autocorrelation. Moran’s I statistic of spatial autocorrelation takes the form described in the equation given below (47–49).


$$I=\frac{N}{\sum_{i}^{N}\sum_{j}^{N}w_{\mathrm{ij}}}\frac{\sum_{I}^{N}\sum_{J}^{N}w_{\mathrm{ij}}(y_{i}-\bar{y})(y_{j}-\bar{y})}{\sum_{i}^{N}{\left(y_{i}-\bar{y}\right)}^{2}}$$

where $w_{\mathrm{ij}}$ are the elements of the spatial weight matrix ( W ) and it is represented as follows:


$$W=(\begin{array}{c} 0\;\;\;\;w_{12}\;\;w_{13}\cdots w_{1N}\\ w_{21}\;\;0\;\;\;\;w_{23}\cdots w_{2N}\\ w_{31}\;\;\;w_{32}\;\;\;0\;\cdots w_{3N}\;\\ \vdots \;\vdots \;\vdots \;\ddots \;\vdots \\ \;w_{N1}\;\;w_{N2}\;\;\;w_{N3}\cdots 0\; \end{array})$$

where N indicates the number of spatial area units.

Geary’s C is another global measure of spatial autocorrelation. Unlike Moran’s I statistics, it is more sensitive to local variations rather than overall patterns, emphasizing differences between neighboring values. Geary’s C > 1 indicates negative spatial autocorrelation (similar values are clustered), Geary’s C < 1 indicates positive spatial autocorrelation (similar values are clustered), and Geary’s C = 1 indicates no spatial autocorrelation (random spatial pattern) and is calculated as:


$$C=\frac{\left(m-1\right)}{2\sum_{i=1}^{N}\sum_{j=1}^{N}w_{\mathrm{ij}}}\frac{\sum_{i=1}^{N}\sum_{j=1}^{N}w_{\mathrm{ij}}{\left((y_{i})-(y)\right)}^{2}}{\sum_{i=1}^{m}{\left(\;y_{i}-\bar{y}\right)}^{2}}$$

with E(C)=1 and m is the number of spatial area units; $\left(y_{i}\right)$ and (y) are the values of the variable of interest at locations $s_{i}$ and $s_{j}$ ; $\bar{y}$ is the mean of the variable y ; $w_{\mathrm{ij}}$ is the spatial weight between locations $s_{i}$ and $s_{j}$ (1 for neighbors, 0 otherwise, and by convention $w_{\mathrm{jj}}=0$ for all j ).

#### 2.3.3 Generalized geo-additive model

Generalized additive models are a practical approach to incorporating spatial smoothing into a model, allowing for the full leverage of existing spatial effects alongside non-linear effects of risk factors in a given problem. The general form of a generalized geo-additive mixed model, for a response variable from the exponential family of distributions, is provided by:


$$g(\mu_{i})=Z_{i}\beta +\sum_{j=1}f_{j}(x_{\mathrm{ji}})+f_{\mathrm{str}}(\cdot )+f_{\text{unst}}(\cdot )$$

where *ɡ*(·) is a link function of the expectation of the response, $Z_{i}$ is the *i*th row of the known design matrix of fixed effects, β is a vector of unknown linear fixed effects, *f_j_* (·) is an unknown non-linear smooth function of the covariate $x_{j}$ , *f_str_* (·) is a structured spatially random effect, *f_unst_* (·) is an unstructured spatial random effect. The above expression can be reformulated for the normally distributed response variable with the identity link function:


$$y_{i}=X_{i}\beta +\sum_{j=1}f_{j}({x^{*}}_{\mathrm{ji}})+u_{i}+v_{i}+\varepsilon_{i}$$

where $u_{i}|(u_{-i},\tau_{u})\sim N({\bar{u}}_{n_{i}},\frac{1}{\tau_{u}n_{i}})$ ; the iCAR model in which the value of the spatial effect $u_{i}$ at location $s_{i}$ is conditionally dependent on the neighboring spatial effect values $u_{-i},$ ${\bar{u}}_{n_{i}}=\frac{1}{n_{i}}\sum u_{-i}$ , where $n_{i}$ is the number of neighbors of a location $s_{i}$ ; and $v_{i}\sim N(0,\frac{1}{\tau_{v}})$ with an independent and identically distributed (i.i.d.) Gaussian model. Finally, $\varepsilon_{i}\sim N(0,\sigma_{\varepsilon }^{2})$ ; random error identically and independently normally distributed, where $\tau_{u}$ and $\tau_{v}$ are the precisions of the structured and unstructured spatial random effects, respectively, and $\sigma_{\varepsilon }^{2}$ is the variance of the random error term.

The response variable is transformed to a normal distribution using the R package called best normalized, which was primarily developed to find a normalizing transformation for a vector of observations (50). Then the inference is based on Integrated Nested Laplace Approximation (INLA); a package developed to approximate Bayesian inference for the Latent Gaussian Model (51, 52). The INLA primarily focuses on individual posterior marginals of the model parameters, rather than estimating the joint posterior distribution of the parameters (43). In many cases, marginal inference is sufficient for making inferences about the model parameters and latent effects, eliminating the need to handle multivariate posterior distributions, which are often difficult to obtain. INLA inference is particularly suited for models that can be expressed as latent Gaussian Markov Random Fields (GMRF), providing significant computational advantages. These advantages make INLA highly versatile and widely applicable across various fields, and it is available through the R-INLA package (53–56).

## 3 Results

### 3.1 Descriptive results

Descriptive statistics for each country, survey year (data collection year), distribution of ANC utilization by countries, and the proportion of ANC utilization among SSA regions were reported in Table 2. A total of 223,155 women were included in the study. The pooled proportion of ANC utilization in SSA countries was 22.15%, with a significant difference compared with country-specific proportions, which range from 0.27% in Rwanda to 76.28% in Zimbabwe (Table 2). There is also sub-regional variation in the proportions of recommended ANC utilization, with the highest proportion observed in the Southern region of SSA countries (71.32%) and the lowest in the Western SSA region (15.61%). Furthermore, the study revealed that in the southern regions of SSA, the highest proportion of ANC utilization, 75.14%, was recorded in South Africa, and the lowest proportion of ANC utilization, 64.63%, was observed in Namibia. In the Central Regions of SSA, the highest proportion of ANC utilization, 47.21%, was recorded in the Democratic Republic of the Congo, and the lowest proportion of ANC utilization, 7.10%, was from Cameroon. In Eastern regions of SSA, the highest proportion of the minimum recommended ANC utilization, 76.28%, was observed in Zimbabwe, and the lowest proportion of minimum recommended ANC utilization, 0.27%, was observed in Rwanda. In the Western regions of SSA, the highest proportion of minimum recommended ANC utilization, 57.11%, was from Togo, and the lowest proportion of minimum recommended ANC utilization, 0.87%, was from Burkina Faso (Table 2).

**Table 2:** Proportion of women of reproductive age who use a minimum recommended ANC contact by country, by region, survey year, and overall proportion across SSA Africa.

S. No.	Country	Survey year	Survey sample	Number of provinces	Number of women (ANC visit)	Proportion of ANC users
1	Angola	2015	6,103	18	475	7.78
2	Benin	2017	8,720	12	782	8.97
3	Burkina Faso	2021	6,211	13	54	0.87
4	Burundi	2016	8,260	18	4,118	49.85
5	Cameroon	2018	5,295	12	376	7.10
6	Chad	2014	10,601	21	3,243	30.59
7	Comoros	2012	1,851	3	902	48.73
8	DC Congo	2013	9,862	11	4,656	47.21
9	Cote D′ Ivoire	2021	4,499	14	177	3.93
10	Ethiopia	2016	7,532	11	2,379	31.59
11	Gabon	2019	3,189	11	434	13.61
12	Gambia	2019	5,045	8	218	4.32
13	Ghana	2022	4,115	16	1,589	38.61
14	Guinea	2018	5,134	8	156	3.04
15	Kenya	2022	7,857	47	339	4.31
16	Lesotho	2014	2,153	10	1,610	74.78
17	Liberia	2019	2,948	5	811	27.51
18	Madagascar	2021	8,324	23	192	2.31
19	Malawi	2015	12,368	3	183	1.48
20	Mali	2018	6,366	9	206	3.24
21	Mauritania	2019	7,669	14	308	4.02
22	Mozambique	2022	5,120	11	95	1.86
23	Namibia	2013	1,948	13	1,259	64.63
24	Niger	2012	7,910	8	2,593	32.78
25	Nigeria	2018	18,217	6	3,800	20.86
26	Rwanda	2019	5,655	5	15	0.27
27	Senegal	2011	7,410	14	165	2.23
28	Sierra Leone	2019	6,308	5	1,388	22.00
29	South Africa	2016	1,460	9	1,097	75.14
30	Tanzania	2022	5,054	30	147	2.91
31	Togo	2013	4,551	6	2,599	57.11
32	Uganda	2016	9,568	15	5,730	59.89
33	Zambia	2018	6,334	10	74	1.17
34	Zimbabwe	2015	9,518	10	7,260	76.28
By region		West Africa		15.61		
		East Africa		24.51		
		South Africa		71.32		
		Central Africa		26.20		
By survey year		Before 2017		37.85		
		After 2017 (included)		9.14		
Overall proportion of SSA				22.15		

**Figure 3:**
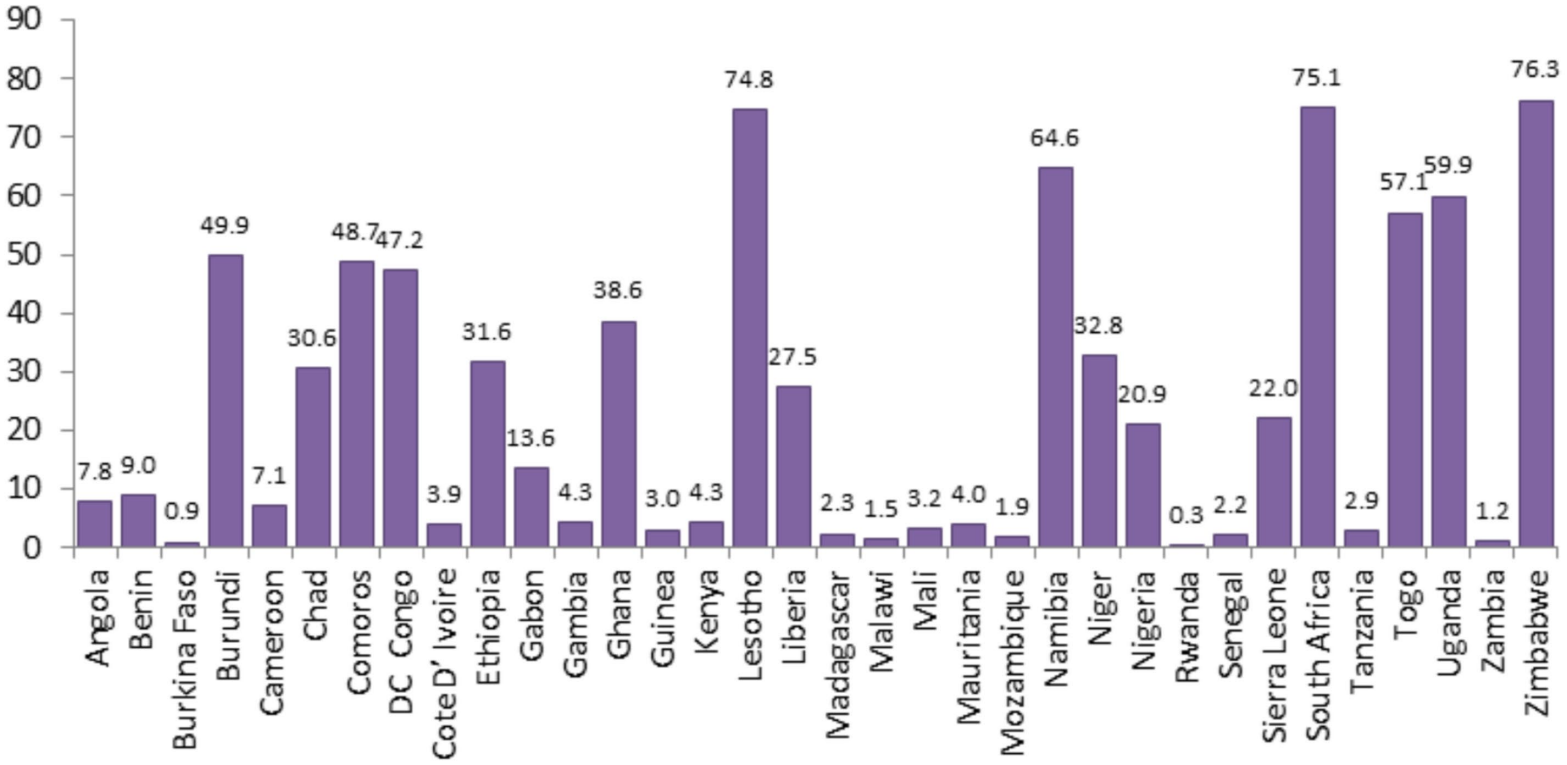
Proportion of minimum recommended ANC utilizations for women of reproductive age across SSA countries.

**Figure 4:**
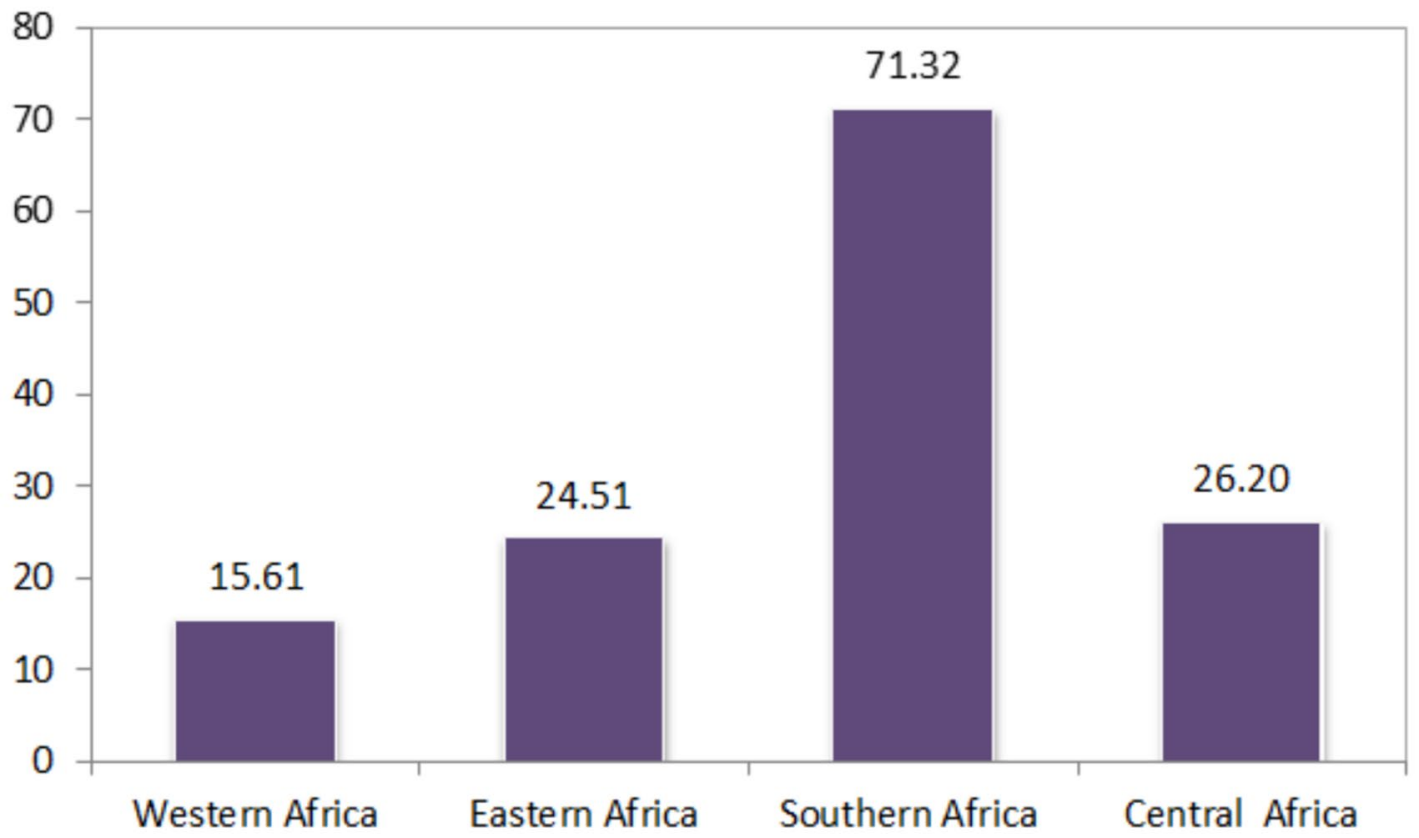
Proportion of minimum recommended ANC utilization for pregnant women of reproductive age across the SSA region.

**Figure 5:**
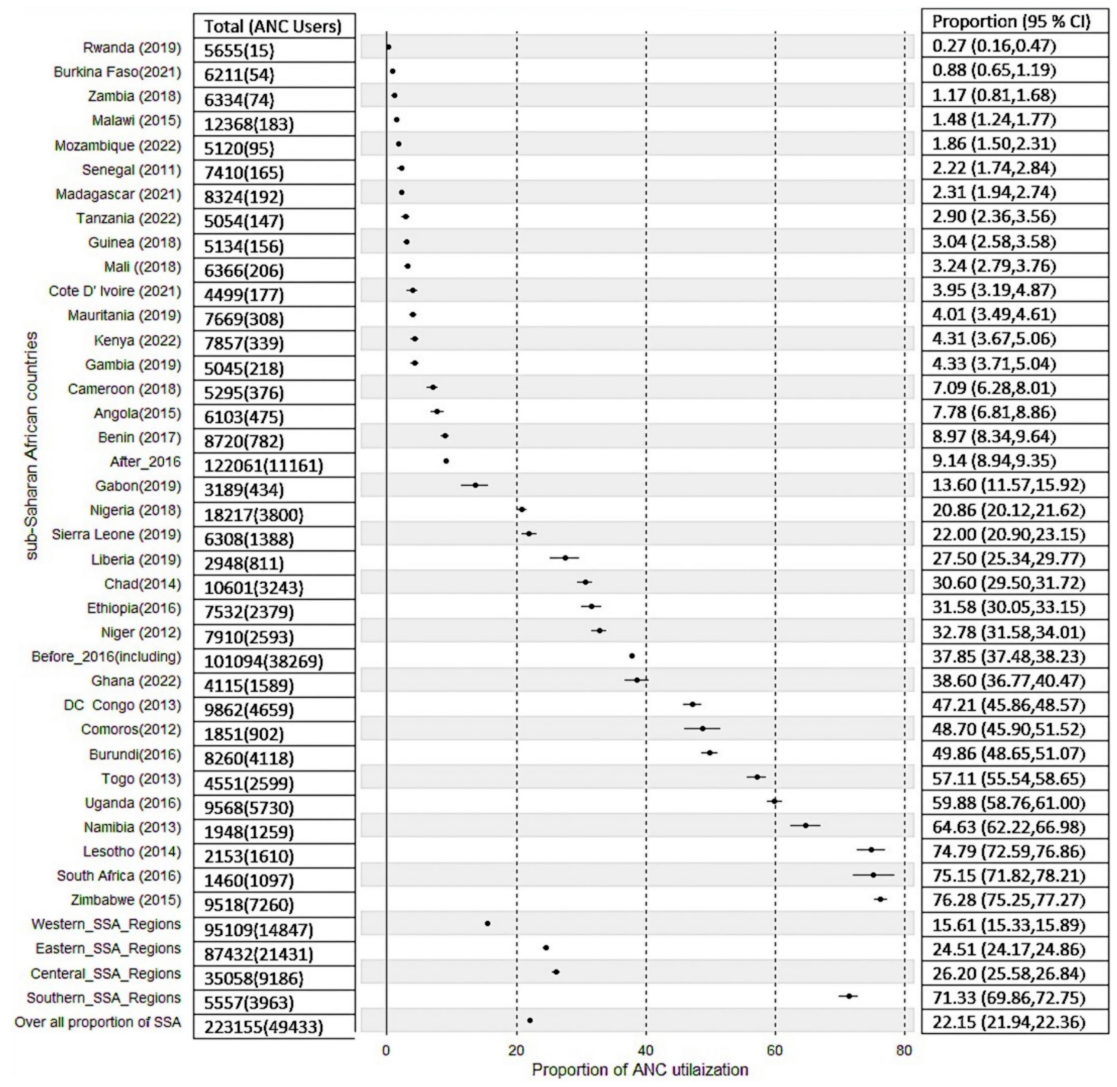
Forest plot of proportion of recommended ANC utilization in SSA countries, and sub-region, and survey year aggregates.

The proportion of minimum recommended antenatal care utilization across sub-Saharan Africa countries showed substantial variation, as illustrated in Figures 3, 4, and the differences were observed in both country-specific and SS African regional level. The regional variations showed that southern SSA regions showed the highest proportions of ANC utilization followed by Central SSA regions and the lowest proportion is observed in western SSA regions. The forest plot of antenatal care utilization proportions for all 34 countries, along with the corresponding 95% confidence intervals (CIs), is displayed in Figure 5, further highlighting the disparities across the sub-Saharan African regions.

### 3.2 Spatial dependency

Table 3 shows the results of the spatial dependency of antenatal care utilization across SSA using Moran’s I and Gear’s C test statistics using different neighborhood assumptions. Moran’s I and Gear’s C test of spatial dependency found that the estimated values are 0.305, 0.679, 0.166, 0.827, 0.129, and 0.869 for Queen, K-nearest, and distance-based neighborhood schemes, respectively. The estimated values of spatial dependency from these two tests are positive, and it is also statistically significant (*p* < 0.001), which indicates the existence of positive spatial autocorrelations of antenatal care utilization.

**Table 3:** Moran’s I and Geary C tests using different neighboring scenarios.

S. No.	Tests	Queen neighborhood			K-nearest neighborhood			Distance-based neighborhood			*p*-value
		Estimate	Expectations	Variance	Estimate	Expectations	Variance	Estimate	Expectations	Variance	
1	Moran’s I Test	0.305	−0.0023	0.00124	0.166	−0.0023	0.0004	0.129	−0.00233	0.0002	<0.001
2	Geary’s C Test	0.679	1.00	0.0015	0.827	1.00	0.0005	0.869	1.00	0.00025	<0.001

### 3.3 Generalized additive model results

**Figure 6:**
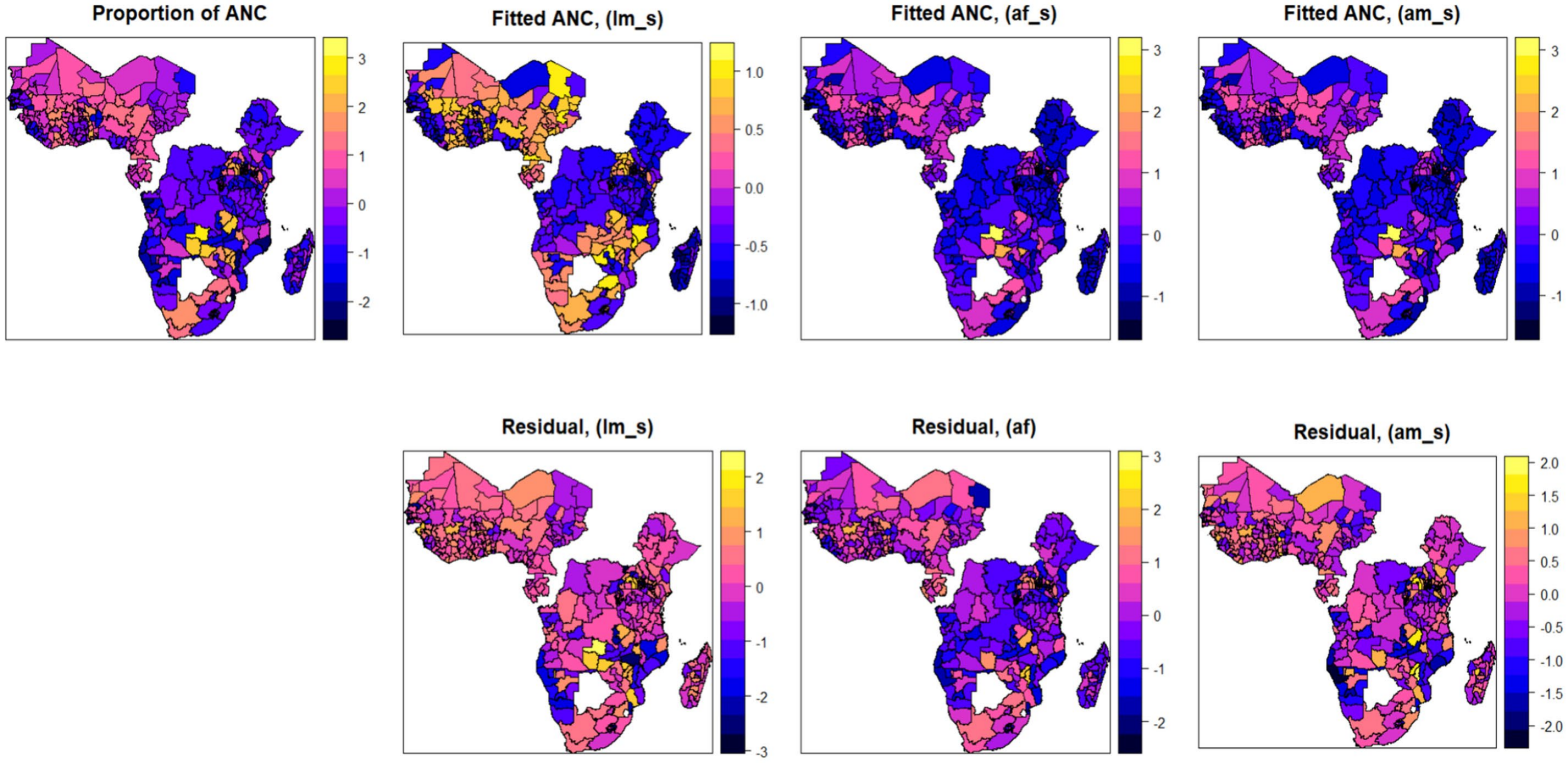
Spatial maps of observed and fitted antenatal care utilization using three different models (linear model, additive with fixed effect, and additive with mixed effect models; with the corresponding residuals).

In this section, we first conducted a preliminary analysis using various statistical models, each based on different assumptions, which helped us to find the best model that fits the data well. Therefore, we initially analyzed the data using three different models by relaxing the functional relationships between the factors and the response variables, which started from the linear model and then introduced a random effect term into the model to assess the predictive performance of each model. We extended the assumptions across three different models to determine their sensitivity under various scenarios. The dataset was restructured in three different ways to evaluate the effects of policy changes and ANC thresholds. Each of the three models was analyzed using these three different data arrangements, focusing on ANC thresholds of four and eight contacts, and the dataset was structured exclusively according to the WHO standard of eight contacts. Then, the three analytical techniques or models were employed to examine how each model responded to the different data configurations. The additive mixed effects model was found to best capture and comprehensively explain the observed data. The detailed results with predictive accuracy, BIC, AIC, and adjusted coefficient of determination can be found in Appendix A. Moreover, Figure 6 also shows the importance of relaxing the linear and fixed effect assumptions of the additive mixed effect model. As shown in the map, three different models, namely, the linear model (fixed effect), the additive model (with fixed effect), and the additive mixed effect model, were employed. The map showed improvements in each model from left to right, and with its respective residuals. From the map, we found that the assumption of employing an additive mixed model has shown better performance, which leads us to use the additive model assumption for better prediction performance. Therefore, by considering the existence of spatial autocorrelation and additive nature of the factors in mixed models, we can apply Bayesian geo-additive mixed model to estimate the model parameters by assuming both additive effect of the risk factor and the existence of spatial effects in the data to fully leverage these two properties in the data (Figure 6).

### 3.4 Bayesian geo-additive modelling approach results

Table 4 presents a comparison of different models using various information criteria. The four models, namely, Bayesian additive models (without random effects), Bayesian additive models [with random effects (i.i.d.)], Bayesian additive models [using Besag-York-Mollié (BYM) model] for both queens and distance-based neighborhood assumption were performed. Based on the information criteria, the Bayesian geo-additive model (Besag-York-Mollié model) with distance-based neighborhood assumption model fitted the data well.

**Table 4:** Comparison of different models using information criteria.

No.	Model	CPO	DIC	WAIC
1	Fixed effect model	−456.22	901.42	48.89
2	Random effect (i.i.d.)	−245.05	477.37	907.25
3	Random effect (structured and unstructured, queens)	−244.90	477.23	480.68
4	Random effect (structured and unstructured, BYM)	**−244.85**	**402.23**	**480.08**

All values in bold are small as compared to others which indicates “Random effect (structured and unstructured, BYM)” is the best model.

**Figure 7:**
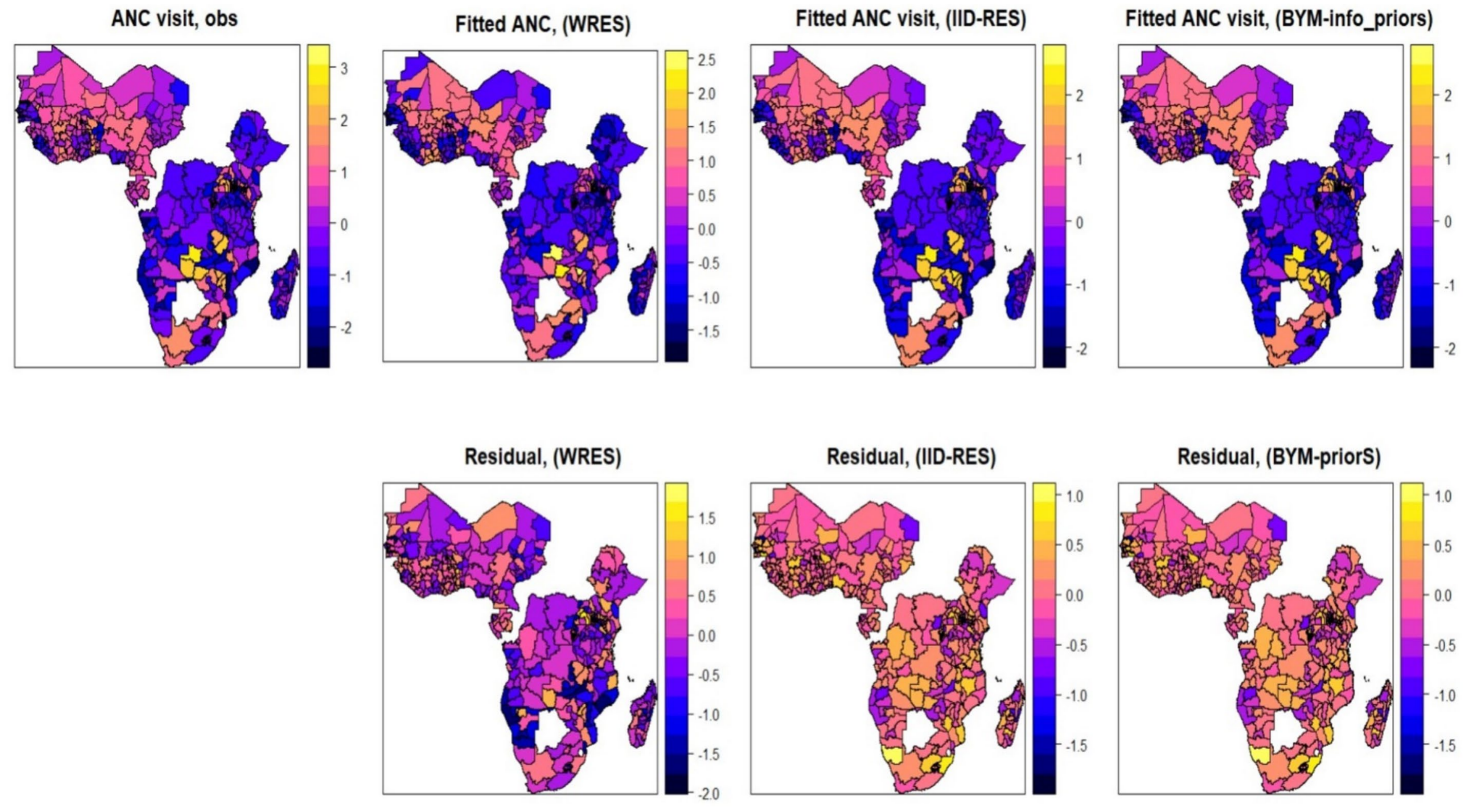
Maps of observed and posterior estimated of proportion antenatal care visits using three different competing Bayesian additive models (without random effect, random effect (with IID), random effect (with BYM) and the corresponding residuals).

Figure 7 shows the spatial distribution of ANC utilization by employing three different spatial additive mixed models. We first fitted the outcome variable using a Bayesian additive model (without a random fixed effect), and then we extended this assumption by assuming a random effect (assuming an i.i.d. Gaussian random effect). Finally, we fitted the Bayesian additive mixed effect model using the Besag-York-Mollié random effect assumption. From the fitted results and the respective residuals, the additive mixed effect model using the Besag-York-Mollié (BYM) random effect model was found to be the best model and chosen as the final model for analysis (Figure 7).

**Figure 8:**
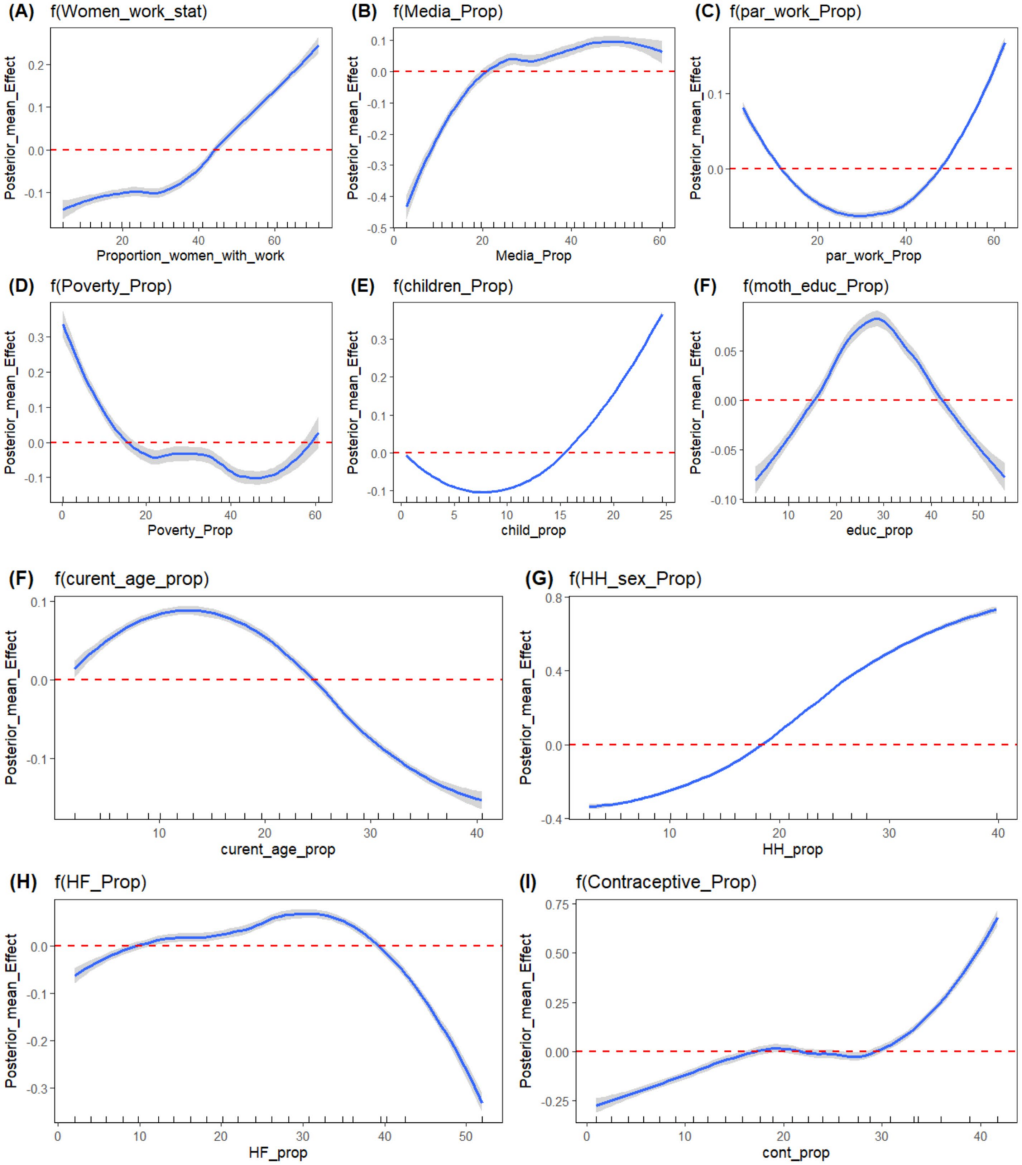
Plots of Posterior mean of non-linear effect of risk factors; **(A)** Women working status, **(B)** Media Exposure, **(C)** Partner’s Working status, **(D)** Proportion of poor HH, **(E)** Total Number of Children, **(F)** Mother’s current age, **(G)** Sex of household head, **(H)** Health Facility, **(I)** Contraceptive use, respectively.

Table 5 shows the estimated posterior means with their corresponding 95% credible interval and standard deviation for the final selected model: Bayesian additive mixed model with Besag-York-Mollié (BYM) mixed effect assumption. Figure 8 also depicts the non-linear effects of risk factors. From the estimated linear fixed effect, the proportion of rural residence, proportion of short birth interval, age at first cohabitation, survey year, and place of delivery (proportion of facility-based delivery) were displayed with their respective posterior means. From the fixed effect factors, the proportion of facility delivery and the survey year have a significant effect on antenatal care utilization. The posterior estimated effect of facility delivery is 1.066 (95% CI: 0.150, 1.980), showing that the proportion of pregnant women who deliver their newborn child in a health facility has a positive effect. This showed that, as the proportion of pregnant women who deliver their newborn child in a health facility increases, the proportion of antenatal care utilization by women also increases. The posterior estimated effect of survey year is −0.945 (95% CI: −1.469, −0.745), indicating that the implementation of the new WHO guideline has had a negative impact on the full utilization of antenatal care contacts. From Appendix B, Kullback–Leibler divergence (KLD) estimates for the fixed effect factors with zero estimated values also indicate the accuracy of estimated fixed parameters using the Gaussian approximation. For other non-linear risk factors, the estimated effects of these factors on the proportion of ANC utilization were estimated using an additive assumption. The effects of these random factors, as shown in Figure 8, indicate non-linear effects. The posterior mean effects, depicted both in the figures and in the table for all non-linear risk factors, show their effect with their 95% credible interval. One of the factors with a non-linear effect is women’s media exposure, which shows an increasing effect, which indicates that as the proportion of women who have media exposure increases, the antenatal care utilization of pregnant women also increases positively. Similarly, the proportion of mothers who are currently working has a positive impact on mothers’ ANC utilization. This shows that, as the proportion of women who are currently working increases, the proportion of recommended ANC utilization coverage also increases. Moreover, the proportion of female-headed households has a positive effect on promoting ANC utilization coverage. This could be attributed to the fact that when a household is headed by women, they may have higher decision-making power and control over pregnancy-related resources. The other non-linear factor, contraceptive usage, has also shown a positive effect on ANC utilization, with a corresponding increase. The ANC utilization coverage in the given region increases as the proportion of contraceptive users increases in that community.

**Table 5:** Estimated posterior means of the parameter with its corresponding 95% credible intervals.

Risk factor (fixed)	Mean	SD	2.5%	50%	97.5%
Intercept	0.524	0.436	−0.333	0.524	1.38
Place of residence	0.0001	0.004	−0.0081	0.00012	0.0071
Birth interval	−0.004	0.015	−0.034	−0.004	0.026
Age at first cohabitation	0.002	0.008	−0.015	0.002	0.019
Place of delivery	1.066	0.466	0.15	1.066	1.980
Survey year	−0.945	0.266	−1.469	−0.745	−0.421

Random effects	Besag (BYM)				
Model hyperparameters	Estimate	SD	2.5%	50%	97.5%
Precision for Gaussian observation	6.74	0.524	5.75	6.73	7.81
Precision for structured	1.94	0.506	1.11	1.88	3.09
Precision for unstructured	2,399.26	2,800	166.86	1,528.9	9,700
Media exposure	22,601	25,300	28,400	15,003.5	89,000
Parent working status	40,783.9	28,400.0	8,508.79	33,647.5	115,000
Mothers working status	36,044.5	27,400	6,395.11	28,795.1	108,000
Poverty index	24,992.6	23,300	3,136.05	18,255.4	86,700.0
Contraceptive usage	10,954.6	21,300	516.09	5071.5	58,600
Total children	19,009.7	34,500	730.0	9,043.7	99,400
Distance from the health facility	27,347.2	31,100	2,398.8	17,919.8	109,000
Mother education	33,922.5	26,100	5,868.9	26,994.5	102,000
Sex_Household head	21,187.1	25,600	2,523.9	17,788.1	93,300
Mothers current age	25,427.5	25,600	2,523.9	17,788.1	93,300
Adjusted R^2^ (R^2^__adj_)			87.5		

Conversely, the proportion of current women’s age, the proportion of very distant health facilities, and the proportion of poor households showed opposite effects to ANC utilization. The proportion of women of current age below the median age has a negative effect on ANC utilization. This shows that as the proportion of young women increases in the region, the proportion of ANC coverage decreases, and this might be due to the younger mother not having the experience for ANC utilization and may know less about pregnancy-related complications. Similarly, the proportion of partners working status has varying effects on ANC utilization. In the beginning, as the proportion of working partners increases, the ANC utilization decreases, and it shifts its direction after some point, at which its effect changes from negative to positive.

The estimated posterior mean of precision of both structured random effect and unstructured random effect is also shown in Table 5. These precisions indicate the variability in the estimated response variable (ANC), which is not explained by both linear and non-linear effect factors considered in the model.

**Figure 9:**
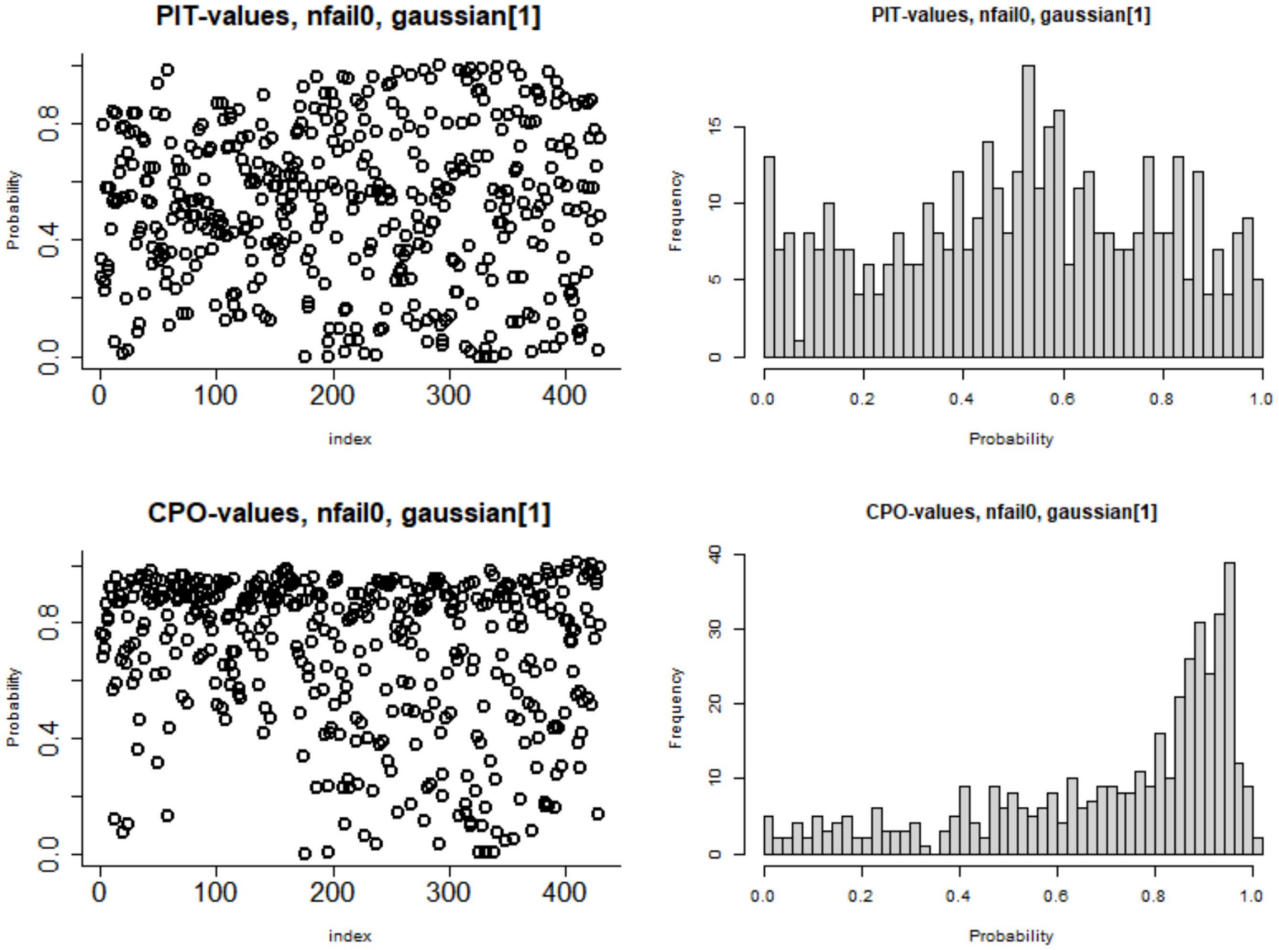
Plots of probability integral transform (PIT) and conditional predictive ordinate (CPO) for the measure of goodness of the Besag-York-Mollié model fit.

Figure 9 presents the Predictive Integral Transforms (PIT) computed for each observation, which measures the probability that a new observation is lower than the actual observed value when the model is fitted using all other observations. For a model to fit the data well, the distribution of PIT values should closely follow a uniform distribution between 0 and 1. This pattern confirms the Bayesian geo-additive model with the Besag-York-Mollié effect model optimally fit to the data.

## 4 Discussion

A total of 223,155 women of reproductive age from 34 countries were used to present the proportions of minimum recommended ANC contacts across SSA countries. The study showed that the overall coverage of the recommended ANC utilization in SSA countries is 22.15%, which is much lower than global coverage, which is about 62%, and even much lower than the coverage in developed countries. The maximum proportion of ANC utilization among 34 countries was observed in Zimbabwe, which is about 76.28%, and this proportion is still lower than the ANC utilization coverage in developed countries, and the minimum proportion (0.27%) is observed in Rwanda. This might have resulted from inequalities in the accessibility of maternal health services, poor or absent of transportation, inequality in the number of healthcare providers, and disparities in access to education. This low maternal antenatal care utilization may also be attributed to regional differences in terms of health services accessibility, adaptation of national policy guidelines related to these recommendations, and socioeconomic status of respondents (57–60). Moreover, the low utilization of antenatal care is attributed to the adherence to the new WHO guidelines, which define adequate ANC as receiving eight or more contacts during pregnancy. In Rwanda, reports indicate that approximately 47% of women attended at least four antenatal care (ANC) visits during pregnancy. However, adherence to and implementation of the updated WHO guideline, which recommends a minimum of eight ANC contacts, remains significantly low, with only about 0.27% of women meeting this standard. This low utilization of ANC is attributed to the delayed adoption of the new WHO guidelines in the country. The Ministry of Health of the Republic of Rwanda introduced the updated national antenatal care (ANC) guidelines referred to as the “National Antenatal Care Guidelines” and began implementation in 2021. This substantial gap underscores the impact of delayed policy approval, which may have hindered progress in aligning with the new ANC guideline and utilization of the updated WHO recommendations (61–63).

In this study, we first analyzed the linear model and extended this assumption with a more complex assumption. We also observed the presence of spatial autocorrelation in the data, which enabled us to incorporate these two properties into an appropriate model. The existence of this spatial dependency in ANC utilization was tested using Moran’s I, Queen’s/Rook’s contiguity, and Geary’s C tests of spatial autocorrelation, which confirmed the presence of spatial dependence in our data. Therefore, from these two separate analyses, it is found that non-linear random effects of factors associated with ANC utilization and spatial autocorrelations are observed in the data. To fully leverage these two properties existed in the data, we employed Bayesian additive mixed model with Besag-York-Mollié (BYM) mixed effect model to see the effects of factors on ANC and the spatial disparities of antenatal care utilization among women with reproductive age who have at least one pregnancy in the last 5 years preceding the survey across 34 sub-Saharan Africa countries.

The underutilization of minimum recommended ANC utilization is mostly observed in western part of SSA region and this lower maternal care utilization may be due to regional differences in health services, compliance of the cares among women, diversity of compassionate care among healthcare providers, sociodemographic status of women and the differences in national minimum ANC contacts policies. Moreover, different regions have distinct cultures; sociodemographic features of women, attitudes toward the usage of ANC, and adaptation of national policy guidelines related to these recommendations. Globally, the vast majority of countries (98%) have national policy guidelines on ANC implementation. Specifically, all countries in the Americas and Southeast Asia have national ANC policies or guidelines in place for implementation. In the African regions, approximately 93–97% of the countries have a national ANC policy or guideline that has been adopted for implementation. Moreover, about 52% of the countries recommended at least four ANC contacts, and 39% of the countries globally recommended at least eight contacts in their national policy (64). Although these standard recommended policies existed in these SSA countries, utilization disparities are observed in the regions of SSA countries. These differences in SSA regions could bring variations in maternal ANC utilization disparities.

The findings of this study showed that women with education had a positive significant effect on recommended ANC utilization, although the effect changes its direction after some point. This finding is similar to that of other previous studies conducted in SSA (65, 66) and Tanzania (67). The study also showed that women’s exposure to mass media has a positive effect on the proportion of women who utilize antenatal care. This finding is consistent with previous studies in sub-Saharan Africa (21), Nigeria (26), and Indonesia (68). The possible explanation for this finding is that providing women with adequate information about maternal health services increases their utilization of such services. Furthermore, targeted media campaigns may be most effective when deigning to reach populations just below the threshold that helps to marginal gains in ANC utilization (69–71).

Moreover, the proportion of women with more than six ever-born children had a significant relationship with recommended antenatal care utilization. The higher the number of children a woman had, the more likely to have recommended antenatal care utilization. This might be due to the reason that women will get experience on how to get pregnancy-related services and their significance for their positive pregnancy health outcomes, and the study is supported by previous studies (65, 72, 73). The study also revealed a detectable non-linear relationship between the proportion of women’s working status and the proportion of ANC utilization. Our findings on the effects of respondents’ poverty index revealed a significant effect on ANC utilization. This indicates that women from households with a below-medium wealth quintile are less likely to utilize ANC service care. This relationship between wealth index and antenatal care utilization is also supported by a study conducted by a previous study (74). Mother’s working status has also been found to have a non-linear effect on ANC utilization. Mothers who are currently working are less likely to have ANC contacts compared to mothers who do not work. The issue may be attributed to various factors such as time constraints, workplace policies, or inadequate support for attending healthcare appointments, and this finding contradicts other previous studies (75, 76). The partner’s working status was also a significant determinant of the utilization of ANC services. This indicates that women whose partners are currently working status were less likely to receive ANC services in the beginning, and then it shifts its effect after some point, and this finding is in agreement with the results of the previous studies. It might be reasoned that the absence of support from a partner may be a hindrance to ANC utilization (77). This finding highlighted the need for intervention strategies for women’s work health policies that allow for flexible schedules, paid maternal leave, and on-site health promotion, which could support ANC utilization among employed mothers (78, 79). The increased and comprehensive utilization ANC services are stalled by insufficient healthcare infrastructure, a shortage of skilled health professionals, and inconsistent availability of medical supplies. Moreover, systemic inefficiencies, such as inadequate governance and limited financial resources, exacerbate existing disparities. It is essential to recognize that understanding the geographical variability in recommended ANC and its determinants is vital for improving maternal and child health interventions (18, 21, 57). Facility-level shortages, such as poor infrastructure, limited medical equipment, and unreliable utilities, significantly hinder the quality and delivery of antenatal care (ANC) services in West Africa. Additionally, geographical barriers play a crucial role, as demonstrated in Burkina Faso, where long distances to health facilities are associated with reduced ANC attendance and facility-based deliveries. These previous studies conform to the current finding, emphasizing the urgent need for strategic investment in healthcare infrastructure and accessibility to improve maternal health outcomes in the region (80, 81).

On the other hand, studies from Central sub-Saharan Africa demonstrate the critical role of cultural sensitization in improving antenatal care (ANC) utilization. In Angola, cultural beliefs, perceptions of care quality, and informal costs contribute to women preferring home births, even in high-risk situations. In Chad, cultural norms significantly hinder the use of ANC, prompting calls for community-based interventions. Cameroon’s experience demonstrates that integrating traditional birth attendants into healthcare systems helps bridge cultural gaps and enhances maternal service access. In the DRC, culturally adapted training for healthcare providers improved the acceptability of ANC services. Although these studies collectively underscore the necessity of context-specific strategies to address cultural barriers and improve maternal health outcomes in the region, the existing underutilization of ANC and spatial disparities in the central SSA region need a strengthened implementation of the campaign for increased utilization of antenatal care (82–85).

### 4.1 Strengths and limitations of the study

This study applied a Bayesian geo-additive modelling approach, which allows for a comprehensive analysis of spatial disparities, and tried to assess the non-linear effects of factors in ANC utilization across sub-Saharan African countries, which allows us to see the cross-country disparities and ANC coverage. Moreover, by using large, nationally representative DHS data across multiple countries, our findings are both robust and policy-relevant. Although this study provides vital insights into ANC utilization, the limitation of this study is the potential recall bias in self-reported ANC visits, where respondents may misremember or underreport visits, leading to inaccuracies in ANC utilization estimates. Future studies should validate self-reported data with health records or facility-based data to minimize recall bias and improve accuracy. This study was unable to evaluate the effectiveness of ANC in relation to key outcomes and distinguish between demand- and supply-side factors influencing ANC utilization. The DHS dataset lacked comprehensive maternal health data like readiness of nearby health facilities, health services, adoption of the new guidelines in the respective country, maternal mortality, and previous pregnancy, and also contained substantial missing values for birth weight. This limited our ability to explore the direct impact of ANC utilization on maternal and neonatal health outcomes. Hence, future research could benefit from incorporating data on both maternal health and neonatal health to explore spatial disparities in antenatal care (ANC) and extend to other medical services.

## 5 Conclusion

The overall coverage of recommended ANC utilization for 34 SSA countries, which is 22.15% ANC utilization based on their recent DHS survey, is far from the global coverage, which is 62%, with a minimum recommended ANC service utilization. Although Zimbabwe has achieved the maximum ANC utilization coverage with 76.28%, this is still a significantly lower proportion than the ANC coverage in developed countries. In this study, we first made a preliminary analysis using linear regression, and then extended it to the non-linear assumption by relaxing the effects of the factor. Moreover, Moran’s and Gearsy’s C test of spatial dependency with different neighborhood structures proved the existence of spatial dependence of the data. To consolidate these two properties in the data, we used a Bayesian geo-additive model with a Besag-York-Mollié (BYM) mixed effect model to examine the spatial disparities in antenatal care utilization among women of reproductive age across sub-Saharan African countries who have at least one pregnancy in the last 5 years preceding the survey. The findings of this study showed the existence of spatial disparities in ANC utilization, and the effect of risk factors is found to have a non-linear effect on ANC utilization across the SSA countries. The lower coverage and inequality of ANC utilizations are influenced by underutilization of healthcare services, economic status, women’s education, coverage disparities, cultural influences, poor/absence of transportation facilities, and media exposure related to healthy reproduction. The increased and comprehensive utilization of antenatal care (ANC) services is stalled by insufficient healthcare infrastructure, a shortage of skilled health professionals, and inconsistent availability of medical supplies. Although there is an effort in context-specific strategies to address cultural barriers and improve maternal health outcomes in the region, the existing underutilization of ANC and spatial disparities need a strengthened implementation of the campaign for increased utilization of antenatal care. The study showed that media exposure, mother’s work status, partner’s working status, age of mother, age at first cohabitation, and place of delivery have significant effects on ANC utilization. Empowering women through different media outlets, strengthening their economic power, easy access to health facilities, and decision-making power increases maternal healthcare service utilization.

## Data Availability

The original contributions presented in the study are included in the article/Supplementary material, further inquiries can be directed to the corresponding author.
