# Supplementary material for: Spatial disparities of antenatal care utilization among pregnant women in sub-Saharan Africa—Bayesian geo-additive modelling approach
Source: Front Public Health. 2025 Jun 27;13:1517724. doi: 10.3389/fpubh.2025.1517724 (PMC12247177; doi:10.3389/fpubh.2025.1517724)
Supplement: Supplementary file 1 [file Table_1.docx]

Appendices

Appendix A: Model Comparison to see the sensitivity of the models using different scenarios.

| Models | Full model | | | | Model with only eight ANC | | | | Model Without survey year | | | |
| --- | --- | --- | --- | --- | --- | --- | --- | --- | --- | --- | --- | --- |
|  | BIC | AIC | R^2^_adj_ | Prediction error  (RMSE/MAE) | BIC | AIC | R^2^_adj_ | Prediction error  (RMSE/MAE) | BIC | AIC | R^2^_adj_ | Prediction error  (RMSE/MAE) |
| Linear model | 1161.7 | 1092.6 | 41.4 | 1.34 | 573 | 516.6 | 31.12 | 1.32 | 1245.3 | 1180.3 | 28.02 | 1.31 |
| Additive model with fixed effect | 1126.0 | 881.7 | 60.8 | 0.83 | 551.7 | 406.4 | 49.2 | 1.30 | 1214.0 | 983.6 | 50.0 | 1.30 |
| Additive model with random effect | 1054.8 | 817.5 | 66.0 | 0.83 | 551.7 | 406.4 | 49.2 | 0.883 | 1207.3 | 978.9 | 50.4 | 0.83 |

Appendix B: Estimated posterior means of parameter with its corresponding 95% credible intervals; and model and kullback-Leibler divergence (KLD) using Bayesian additive mixed model with Besag-York-Mollie (BYM) mixed effect model

| Risk factor(Fixed) | Mean | | SD | | 2.5% | | 50% | | 97.5% | Mode | kld |  |
| --- | --- | --- | --- | --- | --- | --- | --- | --- | --- | --- | --- | --- |
| Intercept | 0.524 | | 0.436 | | -0.333 | | 0.524 | | 1.38 | 0.524 | 0 |  |
| Place of residence | 0.0001 | | 0.004 | | -0.0081 | | 0.00012 | | 0.0071 | 0.0001 | 0 |  |
| Birth interval | -0.004 | | 0.015 | | -0.034 | | -0.004 | | 0.026 | -0.004 | 0 |  |
| Age at First cohabitation | 0.002 | | 0.008 | | -0.015 | | 0.002 | | 0.019 | 0.002 | 0 |  |
| Place of delivery | 1.066 | | 0.466 | | 0.15 | | 1.066 | | 1.980 | 1.066 | 0 |  |
| Survey year | -0.945 | | 0.266 | | -1.469 | | -0.745 | | -0.421 | -0.945 | 0 |  |
| Random Effects: | | | Besag (BYM) | | | | | | | | |  |
| Model hyper parameters | | | estimate | | SD | | 2.5% | | 50% | | 97.5% | Mode |
| Precision for Gaussian observation | | | 6.74 | | 0.524 | | 5.75 | | 6.73 | | 7.81 | 6.71 |
| Precision for structured | | | 1.94 | | 0.506 | | 1.11 | | 1.88 | | 3.09 | 1.78 |
| Precision for unstructured | | | 2399.26 | | 2800 | | 166.86 | | 1528.9 | | 9700 | 454.99 |
| Media exposure | | | 22601 | | 25300 | | 28400 | | 15003.5 | | 89000 | 6182.62 |
| Parent working status | | | 40783.9 | | 28400.0 | | 8508.79 | | 33647.5 | | 115000 | 21706.2 |
| Mothers working status | | | 36044.5 | | 27400 | | 6395.11 | | 28795.1 | | 108000 | 16969.9 |
| Poverty index | | | 24992.6 | | 23300 | | 3136.05 | | 18255.4 | | 86700.0 | 8627.1 |
| Contraceptive usage | | | 10954.6 | | 21300 | | 516.09 | | 5071.5 | | 58600 | 1291.4 |
| Total children | | | 19009.7 | | 34500 | | 730.0 | | 9043.7 | | 99400 | 1788.0 |
| Distance from health facility | | | 27347.2 | | 31100 | | 2398.8 | | 17919.8 | | 109000 | 6574 |
| Mother education | | | 33922.5 | | 26100 | | 5868.9 | | 26994.5 | | 102000 | 15680.2 |
| Sex_of HH-Head | | | 21187.1 | | 25600 | | 2523.9 | | 17788.1 | | 93300 | 7066.7 |
| Mothers current age | | | 25427.5 | | 25600 | | 2523.9 | | 17788.1 | | 93300 | 7066.7 |
| Adjusted R squared (R^2^__adj_) | | | | | | | 87.4 | | | | | |
